# IL-33 levels differentiate tuberculous pleurisy from malignant pleural effusions

**DOI:** 10.3892/ol.2014.2109

**Published:** 2014-05-02

**Authors:** WEI-XIA XUAN, JIAN-CHU ZHANG, QIONG ZHOU, WEI-BING YANG, LI-JUN MA

**Affiliations:** 1 Department of Respiratory and Critical Medicine, The People’s Hospital of Zhengzhou University, Zhengzhou, Henan 450003, P.R. China; 2 Department of Respiratory Medicine, Union Hospital Affiliated to Tongji Medical College, Huazhong University of Science and Technology, Wuhan, Hubei 430022, P.R. China

**Keywords:** interleukin-33, pleural effusion, peripheral blood, tuberculous pleurisy, malignant pleural effusion

## Abstract

Tuberculous pleural effusions (TPEs) and malignant pleural effusions (MPEs) are difficult to differentiate between in certain clinical situations. Interleukin (IL)-33 is a cytokine that participates in inflammatory responses and may have a role in pleural effusions. The present study aimed to investigate the concentrations and potential differential significance of IL-33 in patients with TPE and MPE. IL-33 levels in pleural effusion and serum samples were detected using sandwich enzyme-linked immunosorbent assay in 23 patients with TPE and 21 patients with MPE. The concentration of IL-33 (mean ± standard deviation) in the TPE patients (22.962±0.976 ng/l) was significantly higher than that in the MPE patients (12.603±5.153 ng/l; P<0.001; z=−4.572); however, there was no significant difference in the serum level of IL-33 in the patients with TPE compared with those with MPE (P>0.05). The concentration of IL-33 in the pleural effusions was positively correlated with that in the serum samples in each group (TPE: r=0.563, P=0.05; MPE: r=0.535, P<0.05). The cut-off value of pleural IL-33 for TPE was 19.86 ng/l, which yielded a sensitivity of 0.869, a specificity of 0.905 and an area under the corresponding receiver operating characteristic curve of 0.903. The present study identified that the level of pleural IL-33 is significantly increased in TPEs and may serve as a novel biomarker to differentiate between patients with TPE and MPE.

## Introduction

Tuberculous pleurisy and malignancy are two of the most common causes of pleural effusions, but are difficult to differentiate between in certain clinical situations. Increased pleural permeability and exudates characterizing the inflammatory process lead to the accumulation of pleural fluids and are believed to be key events in tuberculous pleural effusions (TPEs) (1). Certain cytokines and chemokines released by activated inflammatory cells, particularly T lymphocytes and macrophages, are responsible for this process (2). By contrast, malignant pleural effusions (MPEs) are primarily caused by pleural metastasis or lymphatic obstruction and are diagnosed by the presence of malignant cells on the pleura or in the effusions (3). Cytokines, including interferon (IFN)-γ and interleukin (IL)-1, -6, -16 and -17 have shown diagnostic, differential and prognostic significance in TPEs and MPEs (1–3). These findings indicate that the activation of lymphocytes and the release of cytokines may have important roles in the development of TPEs and MPEs.

IL-33 was initially identified as the nuclear factor of high endothelial venules (HEVs) and was termed NF-HEV (4). In 2005, Schmitz *et al* (5) demonstrated that NF-HEV was a novel member of the IL-1 cytokine superfamily, thus NF-HEV was termed IL-33, which is also known as IL-F11 (5). The IL-33 gene is highly enriched in the skin, lung, gastrointestinal tract and brain. IL-33 is expressed by a variety of stromal cells, including epithelial cells, alveolar epithelial cells, endothelial cells, fibroblasts and eosinophilic cells, and its expression is stimulated in response to inflammatory cytokine stimulation following injury or infection (6,7). IL-33 is closely associated with asthma and allergies, autoimmune rheumatic diseases, skin infections, cancer, obesity, type 2 diabetes mellitus and cardiovascular system diseases (8). The primary biological activity of IL-33 is the induction of type 2 helper (Th2) cell immunological responses through Th2 cytokines, including IL-4, IL-5 and IL-13 (9). However, accumulating evidence indicates that IL-33 is also capable of modulating type 1 helper (Th1) cell cytokine responses. Furthermore, IL-33 has been reported to induce potent cluster of differentiation 8 (CD8)-positive T cell responses in response to replicating, prototypic RNA and DNA viruses in mice (10). The binding of IL-33 to DNA, where it acts as a nuclear factor, closely resembles the function of IL-1α (11). The IL-33 precursor can bind to nuclear factor κ-light-chain-enhancer of activated B cells (NF-κB) p65, and IL-1β-induced tumor necrosis factor α is reduced in cells overexpressing the IL-33 precursor (12). Thus, as a cytokine and multifaceted immunomodulator, IL-33 has been proposed to have a role in the pathogenesis of pleural inflammation and effusion. The present study aimed to investigate the presence and potential diagnostic value of IL-33 in TPE and MPE. The concentration of IL-33 in pleural effusion and serum samples was detected in 23 patients with TPE and 21 patients with MPE. The correlation between pleural and serum IL-33 levels was analyzed and the diagnostic value of IL-33 was assessed using receiver operating characteristic (ROC) curves.

## Materials and methods

### Study design

The present study was performed at the Union Hospital Affiliated to Tongji Medical College (Huazhong University of Science and Technology, Wuhan, China) and the Wuhan City Tuberculosis Prevention and Control Institute (Wuhan, China). The procedures were approved by the medical ethics committees of the two hospitals. All patients and family members were fully informed about the procedure and signed consent documents. Hospitalized patients with TPE and MPE were screened according to inclusive and exclusive criteria between December 2009 and June 2011. A total of 23 patients with TPE (17 male, female; age, 19–70 years; median age, 45 years) and 21 patients with MPE (10 male, 11 female; age, 8–84 years; median age, 51 years) were selected for the present study. The patients with MPE were suffering from lung cancer (n=18), breast cancer (n=2) or lymphoma (n=1).

### Diagnostic criteria

TPE was diagnosed as pleural effusions meeting any of the following criteria and the exclusion of other causes: (i) The detection of acid-fast bacilli in the pleural effusion examination and/or granuloma-like alterations in the pleural biopsy samples, and the exclusion of pleurisy from other causes; (ii) the identification of exudate pleural effusions, assessed using Light’s criteria (13). Light’s criteria suggest that a pleural effusion is an exudate if lymphocytes are the major cell type in the pleural effusion, the pleural effusion has an adenosine deaminase (ADA) concentration >40 U/l, the tuberculin test is positive, the pleural effusion is absorbed and if the clinical symptoms are reduced with anti-tuberculosis treatment.

The diagnosis of MPE should be made only with the pathological diagnosis of a primary malignancy, radiological or clinical evidence of pleural effusions and cytological/pathological diagnosis of metastatic tumor cells. Moreover, imaging results should be in accordance with primary bronchogenic lung cancer complicated by pleural effusion. Transbronchial or thoracoscopic lung biopsy should pathologically diagnose lung cancer, and the cast-off cells from the pleural effusion should be detected as metastatic tumor cells.

### Exclusion criteria

Patients with any of the following criteria were excluded from the present study: (i) Invasive pleural cavity inspection and/or treatment or chest trauma three months prior to admission; (ii) treatment with anticancer or antituberculosis therapy and the use of glucocorticoid, non-steroidal anti-inflammatory drugs or immunosuppressants; (iii) the detection of metastatic tumor cells in the pleural effusion; or (iv) no diagnosis of pleural effusion.

### Sample collection and IL-33 measurement

Pleural effusion samples were obtained using a standard thoracentesis procedure within 24 h of the patients being hospitalized. Serum samples (20 ml) were obtained from venous blood collected at the same time as the pleural effusion samples. Pleural effusion and serum samples were centrifuged with 500 U/ml heparin at 200 × g for 5 min. The supernatants were collected and frozen at −80°C until required. IL-33 concentration was detected using an enzyme-linked immunosorbent assay kit (Biolegend Inc., Chicago, IL, USA) according to the manufacturer’s instructions. All samples were analyzed twice.

### Statistical analysis

Data are presented as the mean ± standard deviation. The differences between the serum and pleural IL-33 levels, and between the IL-33 levels in the TPE and MPE groups were compared using independent sample t-tests for normally distributed data and Mann-Whitney U or Kruskal-Wallis rank sum tests for non-normally distributed data. Correlations were determined using Spearman’s rank correlation analysis. The diagnostic accuracies of serum or pleural IL-33 levels for discriminating between TPE and MPE were investigated using ROC curve analysis. Data were analyzed using SPSS version 5.0 (SPSS, Inc., Chicago, IL, USA) and SigmaPlot 10.0 (Systat Software, Inc., Chicago, IL, USA). P<0.05 was considered to indicate a statistically significant difference.

## Results

### IL-33 concentrations in pleural effusions

**Figure 1:**
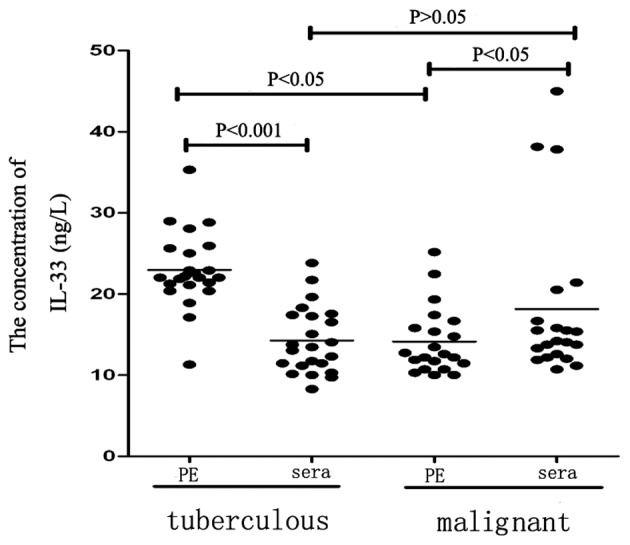
Interluekin (IL)-33 concentration in tuberculous pleural effusions (TPEs) and malignant pleural effusions (MPEs).

Results showed that IL-33 was present in the patients with TPE and MPE. The IL-33 concentration in the pleural effusion (22.96±0.98 ng/l) was significantly higher than that in the corresponding concentration of serum (14.27±0.86 ng/l; P<0.01; Table I; Fig. 1) in the patients with TPE. Furthermore, the concentration of IL-33 in the pleural effusion (12.60±5.15 ng/l) was significantly lower than that in the corresponding serum concentration (14.20±6.22 ng/l; P<0.05; Table I) in the patients with MPE. The concentration of IL-33 in the pleural effusion of the patients with TPE (22.96±0.98 ng/l) was significantly higher than that in the patients with MPE (12.60±5.15 ng/l; P<0.01); however, the concentration of serum IL-33 in the patients with TPE was not significantly different to that in the patients with MPE (P>0.05). Statistically significant differences were identified in the pleural effusion/serum gradient and ratio between TPE and MPE (P<0.01).

### Serum IL-33 level is correlated with pleural IL-33 level in the patients with TPE and MPE

**Figure 2:**
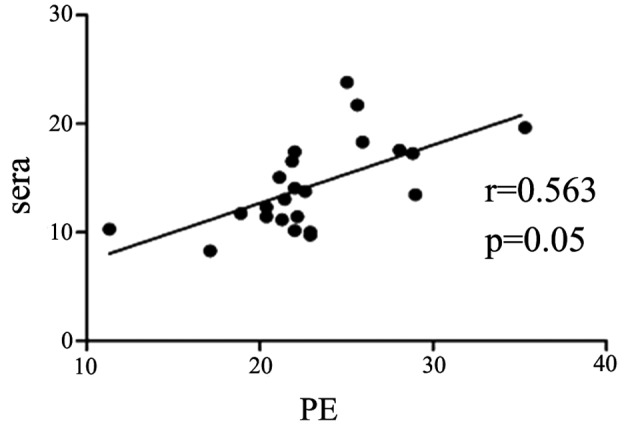
Correlation between serum and pleural IL-33 concentration in tuberculous pleural effusions (TPEs). IL, interleukin.

**Figure 3:**
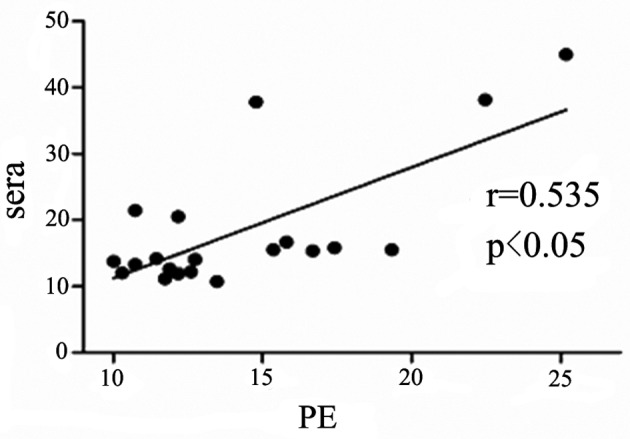
Correlations between serum and pleural IL-33 concentration in malignant pleural effusions (MPEs). IL, interleukin.

The concentration of IL-33 in the pleural effusions was positively correlated with that in the serum samples in the patients with TPE and MPE (r=0.56, P=0.05; and r=0.54, P<0.05; Figs. 2 and 3, respectively).

### IL-33 level distinguishes between patients with TPE and those with MPE

The cut-off value of pleural IL-33 for the diagnosis of TPE was 19.86 ng/l, therefore patients with IL-33 concentrations higher than this threshold had a high probability of being diagnosed with TPE. The value for the area under the corresponding ROC curve (AUC) was 0.903, the 95% confidence interval was 0.80–1, the corresponding sensitivity and specificity were 86.96 and 90.48% (P<0.01), the positive likelihood ratio (+LR) was 9.13 and the negative likelihood ratio (−LR) was 0.13. The concentrations of pleural IL-33, but not serum IL-33, differentiates TPE and MPE (AUC=0.903 and P<0.001 for TPE; and AUC=0.617 and P=0.184 for MPE).

## Discussion

The present study aimed to investigate the concentration and potential diagnostic significance of IL-33 in TPE and MPE. The level of IL-33 in pleural effusion and serum samples obtained from patients with MPE and TPE were analyzed. The present study identified that the level of IL-33 was increased in the pleural effusions and sera of the patients with MPE and TPE. Furthermore, the pleural IL-33 level was found to be higher than the serum IL-33 level in the patients with TPE. Moreover, the concentration of pleural IL-33 in the patients with TPE was observed to be significantly higher than that in the patients with MPE, which indicated that IL-33 may serve as a biomarker for distinguishing between TPE and MPE. The findings of the present study are consistent with those of the study by Lee *et al* (14), which found that pleural and serum IL-33 levels were higher in patients with TPE compared with those patients with other types of pleural effusion, and that pleural IL-33 is of diagnostic significance in distinguishing between TPE and other types of pleural effusion.

IL-33 belongs to the IL-1 subfamily and was formerly termed IL-1F11. Neutrophil proteinase 3, neutrophil elastase and cathepsin G are capable of truncating precursor IL-33, generating IL-33 with different N-termini and varying levels of activity (15). Thus, through processing the IL-33 precursor, neutrophil enzymes generate active IL-33 isoforms with varying levels of activity. Neutrophils are activated in various stages of pleural tuberculosis infection, and thus may contribute to the elevated level of IL-33 in pleural effusions (16). However, it has yet to be elucidated which cells in the pleura or effusions produce the most IL-33 precursor, and whether the IL-33 gene is overexpressed in TPE.

Though binding with its receptor IL-33Rα, IL-33 exerts immunoregulatory capabilities, including inducing Th2 immune responses, and Th1 responses in certain situations (9–12,17). The cytokine balance is primarily Th2-dominant in MPE, while Th1 responses dominate TPE (18). In situations other than MPE or TPE, IL-33 induces the production of IFN-γ, which enhances the Th1 responses (19–21). IFN-γ directly regulates the innate immunity against tuberculosis, therefore IL-33 may modulate and enhance host immunity through the induction of IFN-γ in tuberculous pleurisy (22–25). IL-33 may also regulate the functional status of macrophages, which may regulate infectious and tumor immunity (26). Furthermore, IL-33 has been reported to be expressed in endothelial cells in normal organs, but not in tumor tissues (27,28). IL-33 may have a significant role in tumor angiogenesis and immunosurveillence. IL-33 has been found to enhance the activity of CD8^+^ T cells, NK cells and other tumor killer cells, therefore IL-33 may also have a role in tumor immunity or immunotherapy (10,11,20). These hypotheses require further investigation.

**Figure 4:**
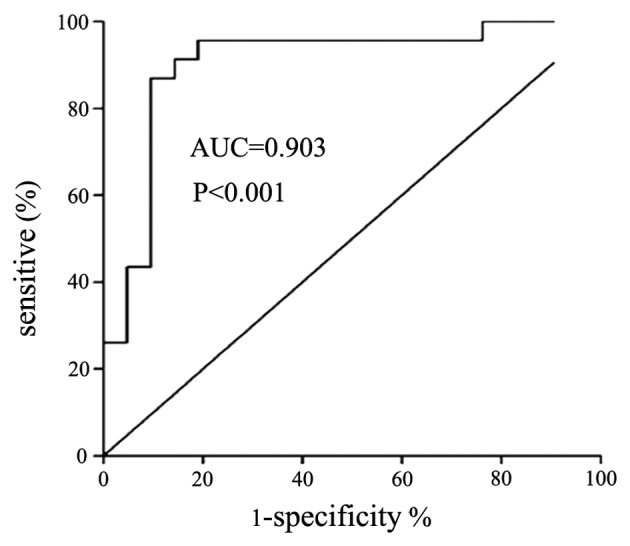
Diagnostic value of pleural IL-33 in tuberculous pleural effusions (TPEs) assessed using receiver operating characteristic (ROC) curve analysis. IL, interleukin; AUC, area under the ROC curve.

**Figure 5:**
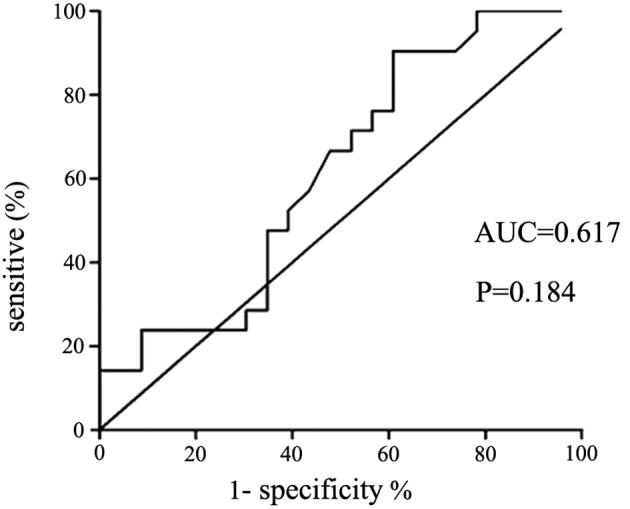
Diagnostic value of serum IL-33 in tuberculous pleural effusions (TPEs) assessed using receiver operating characteristic (ROC) curve analysis. IL, interleukin; AUC, area under the ROC curve.

In the present study, the pleural effusion-serum IL-33 gradient and the pleural effusion-serum IL-33 ratio were observed to be significantly increased in the patients with TPE compared with those with MPE. This may be due to the sharper elevation of IL-33 in the TPE patients compared with the MPE patients, and may reflect the hyperinflamed pleural cavity following tuberculosis infection (16). This also indicates that pleural IL-33 may be a better candidate than serum IL-33 for the diagnosis of TPE, consistent with the overall findings of the present study (Fig. 4 and 5). There are various other biomarkers specific for TPE, including IFN-γ and ADA, which have been standardized as diagnostic criteria for TPE. Lee *et al* (14) compared the diagnostic accuracy of pleural IL-33, IFN-γ and ADA, which yielded AUCs of 0.74, 0.97 and 0.95, respectively. The diagnostic significance of IL-33 may increase if combined with other parameters.

There are several limitations to the present study. Firstly, the reliability of this study is limited by its sample size. Compared with the study by Lee *et al* (14), the present study showed a higher AUC of IL-33 in differential diagnosis (0.903 vs. 0.74). Thus a larger sample size is required for a more accurate assessment of IL-33 in distinguishing between TPE and MPE. Secondly, the present study does not provide direct evidence of IL-33-producing cells and whether the IL-33 gene is overexpressed in TPE. Immunological methods, including flow cytometry, western blot analysis and immunostaining, may be used in future investigations to identify the IL-33-producing cells. Thirdly, the concentrations of IL-33 have not been followed in the treatment of MPE or TPE, which may provide evidence to the correlation between IL-33 and the pathobiology of MPE and TPE.

In conclusion, the present study showed that the pleural IL-33 level is significantly elevated in patients with TPE and may serve as a novel biomarker to differentiate between TPE and MPE. Further investigations are required to identify the specific mechanisms and effects of IL-33 release in TPE and MPE.

## Figures and Tables

**Table I:** IL-33 expression in the pleural effusion and serum and their differences and ratios in patients with tuberculous pleural effusion and malignant pleural effusion.

IL-33	Tuberculous	Malignant	P-value
Pleural effusion, ng/l	22.92±0.98	12.60±5.15	<0.01
Serum, ng/l	14.27±0.86	14.20±6.22	>0.05
Pleural effusion-serum gradient, ng/l	8.69±0.82	−1.31±6.56	<0.01
Pleural effusion-serum ratio, ng/l	1.68±0.77	0.86±0.53	<0.01

Data are presented as the mean ± standard deviation.
